# Transcriptome Analysis of mRNA and lncRNA Related to Muscle Growth and Development in Gannan Yak and Jeryak

**DOI:** 10.3390/ijms242316991

**Published:** 2023-11-30

**Authors:** Yali Wei, Dashan Guo, Yanbin Bai, Zhanxin Liu, Jingsheng Li, Zongchang Chen, Bingang Shi, Zhidong Zhao, Jiang Hu, Xiangmin Han, Jiqing Wang, Xiu Liu, Shaobin Li, Fangfang Zhao

**Affiliations:** Gansu Key Laboratory of Herbivorous Animal Biotechnology, College of Animal Science and Technology, Gansu Agricultural University, Lanzhou 730070, China; weiyl@st.gsau.edu.cn (Y.W.); guods@st.gsau.edu.cn (D.G.); shibg@gsau.edu.cn (B.S.);

**Keywords:** Jeryak, Gannan yak, mRNA–lncRNA, longissimus dorsi, growth performance

## Abstract

The production performance of Jeryak, resulting from the F1 generation of the cross between Gannan yak and Jersey cattle, exhibits a significantly superior outcome compared with that of Gannan yak. Therefore, we used an RNA-seq approach to identify differentially expressed mRNAs (DEMs) and differentially expressed lncRNAs (DELs) influencing muscle growth and development in Gannan yaks and Jeryaks. A total of 304 differentially expressed lncRNAs and 1819 differentially expressed mRNAs were identified based on the screening criteria of |log 2 FC| > 1 and FDR < 0.05. Among these, 132 lncRNAs and 1081 mRNAs were found to be down-regulated, while 172 lncRNAs and 738 mRNAs were up-regulated. GO and KEGG analyses showed that the identified DELs and DEMs were enriched in the entries of pathways associated with muscle growth and development. On this basis, we constructed an lncRNA–mRNA interaction network. Interestingly, two candidate DELs (MSTRG.16260.9 and MSTRG.22127.1) had targeting relationships with 16 (*MYC*, *IGFBP5*, *IGFBP2*, *MYH4*, *FGF6*, etc.) genes related to muscle growth and development. These results could provide a basis for further studies on the roles of lncRNAs and mRNAs in muscle growth in Gannan yaks and Jeryak breeds.

## 1. Introduction

Yak is a specialty germplasm resource mainly distributed in Tibet, Sichuan, Yunnan, and Gansu and is an important resource for local herders to survive. However, the slow growth of yak, slow muscle development, low fat deposition efficiency, and poor meat tenderness significantly impede the production performance and economic benefits of yak. Jeryak is a hybrid resulting from the crossbreeding of Jersey cattle and Gannan yak. Phenotypic analysis revealed that under the same feeding conditions, Jeryak exhibited significantly superior production performance in terms of body weight, shoulder height, body length, and chest circumference compared with Gannan yak [1,2]. Hence, it is of great relevance to study the molecular mechanism of muscle growth and development in Jeryak and Gannan yak.

The developmental process of skeletal muscle determines the yield and quality of livestock and poultry meat products [3]. The growth and development of muscle are determined by the number of pre-birth fibers of the myofiber type, while the hypertrophy process of the area of myofibers mainly occurs in the post-natal period [4,5,6]. Muscle fibers are the fundamental building blocks of muscles, and their properties have a direct impact on animal meat production and quality [7]. The diameter, density, area, and type of muscle fibers are crucial indicators that reflect the characteristics of muscle fibers [8,9]. It has been confirmed that an increase in muscle fiber area and diameter leads to a reduction in muscle tenderness and tensile strength; on the contrary, a decrease in muscle fiber area and diameter leads to increased meat tenderness [10]. In addition to myofiber area and diameter, the myofiber type also affects muscle growth and development [11,12]. Furthermore, the expression of specific mRNAs in skeletal muscle tissues, including *IGFs* [13], *PAXs* [14], *MRFs* [15], and *MEF2* [16], exerts a significant influence on both muscle development and meat quality.

Long non-coding RNA (lncRNA), as one key member of non-coding RNAs, has been extensively investigated in recent years to substantiate its regulatory role in muscle growth and development. Among the various approaches employed to explore lncRNA targets, cis-regulation and trans-regulation of genes have emerged as commonly utilized methods [17,18]. For instance, in skeletal muscle, lncMAAT exhibits cis-regulation to enhance the expression of the Mbnl1 gene while concurrently exerting trans-regulation control over *SOX6* to suppress miR-29b expression through a regulatory module, thereby delaying muscle atrophy [19]. Additionally, lncRNAs serve as molecular sponges for microRNAs, forming a bond with miRNAs to alleviate the suppressive impact of miRNAs on target genes. Through its role of molecular sponge, lncIRS1 absorbs the miR-15 family, thereby activating the IGF1-PI3K/AKT pathway and exerting an impact on muscle atrophy [20]. An additional instance involves the functioning of lncMD, which enhances bovine myoblast differentiation by increasing the expression of *IGF2* through the adsorption of miR-125b [21]. It has also been discovered that lncRNAs exert a regulatory function in the process of muscle regeneration following injury. For that instance, lncRNA MAR1 exhibits a potential positive impact on skeletal muscle hypertrophy and may also contribute to mitigating aging-related muscle atrophy [22]. During the pre-natal and early post-neonatal stages in porcine skeletal muscle, lncRNA MEG3 demonstrates elevated levels of expression, and it plays a crucial part in the development and upkeep of early skeletal muscle [23]. Many researchers have identified the crucial genes that influence muscle development through the comprehensive sequencing of lncRNAs across distinct species. For example, Yang et al. sequenced the longest dorsal muscles of Leiqiong and Lufeng cattle and found 12 genes regulated by DE lncRNA cis; they suggested that the *DKK1* and *SIGMAR1* genes may play a crucial role in muscle development regulation [24]. However, limited research has been conducted regarding the influence of lncRNAs on the process of muscle growth and development in Jeryaks.

Therefore, in this study, adult Gannan yaks and Jeryaks were selected to investigate the regulatory mechanisms of the differences in muscle fiber tissue characteristics between Gannan yaks and Jeryaks by using HE staining, and fast and slow muscle fluorescence staining. Further, RNA-seq was used to study the expression of lncRNA in the dorsal longissimus muscle tissues of the two breeds, and the mRNA–lncRNA interaction network was constructed to identify the key genes for muscle growth and development. These results could provide a basis for further excavation of important lncRNAs in the skeletal muscle development of Jeryaks, and subsequent characterization and functional studies.

## 2. Results

### 2.1. Observable Disparities in Muscle Fibers among Various Breeds of Beef Cattle

**Figure 1:**
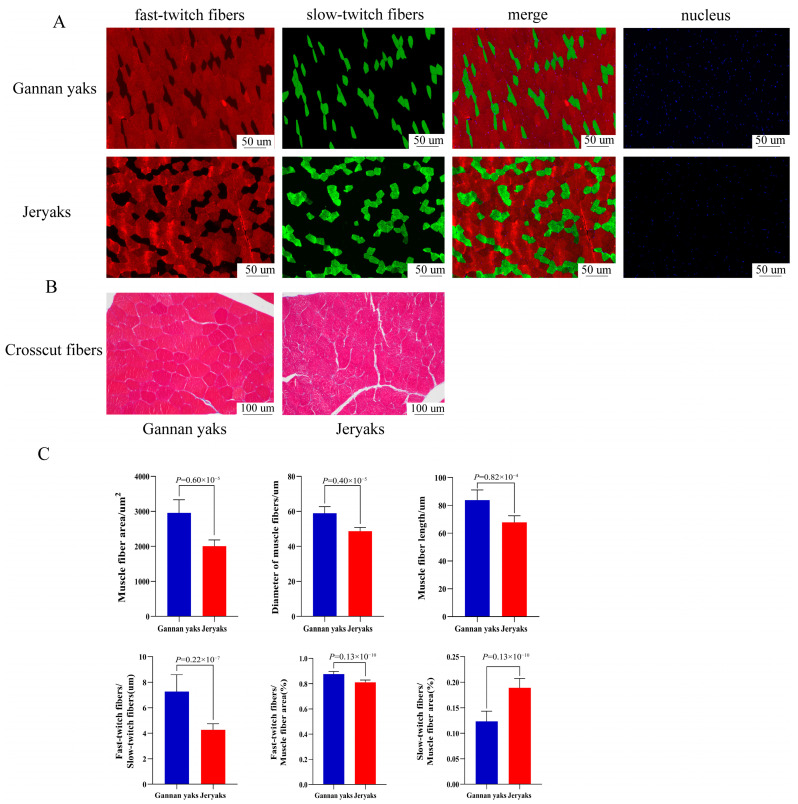
Fast/slow muscle fluorescence staining and paraffin section HE staining of muscle tissue. (**A**) Red represents fast muscle fiber; green represents slow muscle fiber; and blue represents the nucleus of the muscle cell. (**B**) Red represents a single muscle fiber. (**C**) Comparison of myofiber characteristics of different breeds of cattle. Gannan Yak in blue and Jeryak in red.

First, we performed fast and slow myofiber fluorescence staining (Figure 1A) and HE staining (Figure 1B) on Gannan yaks and Jeryaks. The results showed that there were highly significant differences in myofiber characteristics between Gannan yaks and Jeryaks. IPP (Image-Pro Plus 6.0) software analysis showed that the average myofiber area, myofiber diameter, and myofiber length of Gannan yaks were significantly greater than those of Jeryaks (*p* < 0.01), The ratio of fast-to-slow muscle fibers and the ratio of fast muscle fibers to muscle fiber area were significantly greater than in Jeryaks (*p* < 0.01), while the ratio of slow muscle fibers to muscle fiber area was significantly smaller than that of Jeryaks (*p* < 0.01) (Figure 1C).

### 2.2. Sequencing Data Summary

**Figure 2:**
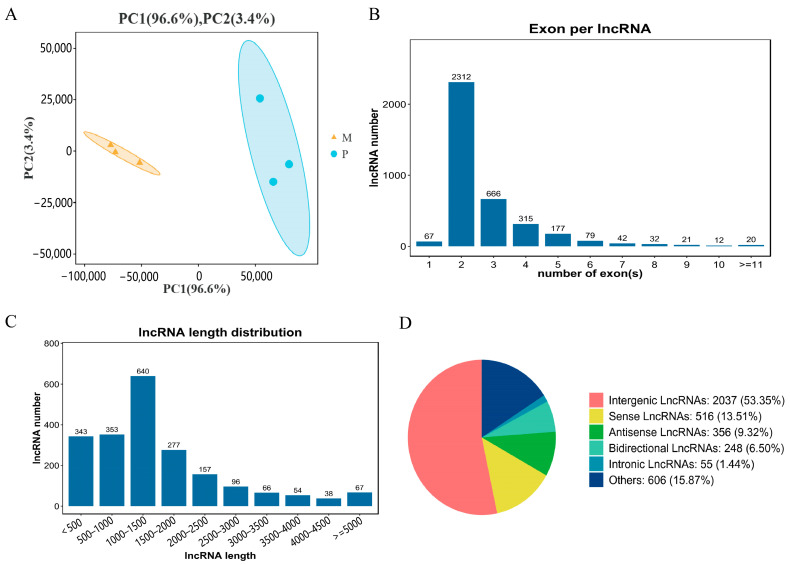
Analysis of lncRNA sequencing data. (**A**) Principal component analysis (PCA) of sample.The figure shows Gannan yaks (M1, M2, M3) in orange and Jeryaks (M1, M2, M3) in blue. (**B**) Exon number distribution of novel lncRNAs. (**C**) Length distribution of the novel lncRNAs. (**D**) Classification of novel lncRNAs.

For this study, six sequencing libraries were constructed, and the cDNA libraries of Gannan yak (M) and Jeryak (P) were sequenced using the Illumina NovaSeq 6000 sequencing platform. On average, each group obtained raw data of 86,609,737 and 91,797,352 reads. On average, each group obtained 86,111,288.67 (99.41%) and 88,349,502.1 (99.50%) high-quality sequences (clean reads) after eliminating the splice sequences and considering N (modular base) reads and reads with low quality at the 3’ end of the reads. Additionally, all six sequenced libraries exhibited Q30 values exceeding 91%, indicating excellent sequencing quality. Furthermore, the GC/AT content was approximately 60% in alignment with the theoretical genome distribution (Table 1). The comparison between pristine reads and the yak’s reference genome revealed a genome comparison rate of over 70% for each sample, demonstrating a favorable comparison effect (Table 2). More than 55% of the reads were uniquely mapped to the genome, indicating a high level of accuracy and reliability in the pristine reads, which are suitable for further analysis. We identified a substantial number of lncRNAs through comprehensive screening and filtering. PCA analysis showed good reproducibility within sample groups and large differences between groups (Figure 2A).

Among these, a total of 3082 lncRNA were recently identified, exhibiting an average size of 1165 base pairs. The largest percentage (30.61%) was observed in the lncRNA category, with a length ranging from 1000 to 1500 base pairs (Figure 2C), while approximately 61.77% of lncRNA consisted of two exons (Figure 2B). Additional categorization of the identified lncRNA indicated that intergenic lncRNA constituted 53.35%, while sense lncRNA made up 13.51%, antisense lncRNA comprised 9.32%, bidirectional lncRNA represented 6.5%, intronic lncRNA constituted 1.44%, and other lncRNAs accounted for 15.87% of the total (Figure 2D).

### 2.3. Expression of Long Non-Coding RNA (lncRNA) and Messenger RNA (mRNA) at the Genomic Level

**Figure 3:**
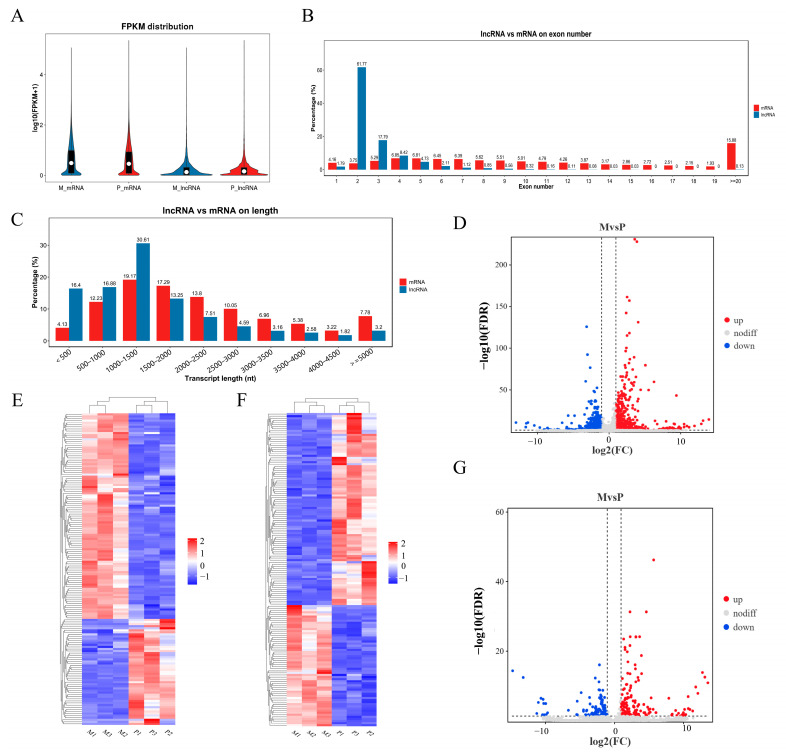
Genomic expression of lncRNA and mRNA. (**A**) The expression of lncRNA and mRNA. (**B**,**C**) Comparison of length and exon count between lncRNA and mRNA. (**E**,**F**) Cluster heatmap of DEMs and DELs. The FPKM array was centered and scaled in the row direction using R package pheatmap (v1.0.8). Red indicates higher expression, and blue represents lower expression. The log10 (FPKM + 1) value was converted (scale number) and clustered. (**D**,**G**) Volcano plot of DEMs and DELs.

With an analysis of gene structure and expression, we aimed to identify discrepancies between lncRNA and mRNA obtained with RNA-seq. Based on the FPKM value, our findings revealed comparatively elevated expression levels for both mRNA and lncRNA (Figure 3A). The average number of exons in mRNA was higher (23.04) compared with lncRNA (average 2.8). In addition, 86.78% of messenger RNA contained four or more exons, whereas 81.35% of long non-coding RNA had three or fewer exons (as shown in Figure 3B). The length of mRNA (averaging 2291 bp) was significantly greater than that of lncRNA (averaging 1363 bp) (Figure 3C). In the longissimus dorsi muscle of Gannan yak and Jeryak, the measurement was conducted on a combined count of 3743 lncRNAs and 16,143 mRNAs. Using Gannan yak as the reference group, a total of 304 differentially expressed lncRNAs (DELs) and 1819 differentially expressed mRNAs (DEMs) were identified. Among these, 132 lncRNAs and 1081 mRNAs were found to be down-regulated, while 172 lncRNAs and 738 mRNAs were up-regulated (Figure 3D,G). Furthermore, a separate cluster analysis was conducted on DELs and DEMs, revealing that the identical sets of DELs and DEMs were grouped together, thus confirming the precision and dependability of the samples (Figure 3E,F). Tables S1 and S2 show the DEMs and DELs, respectively.

### 2.4. Performing GO and KEGG Analyses on Differentially Expressed Genes (DEGs)

**Figure 4:**
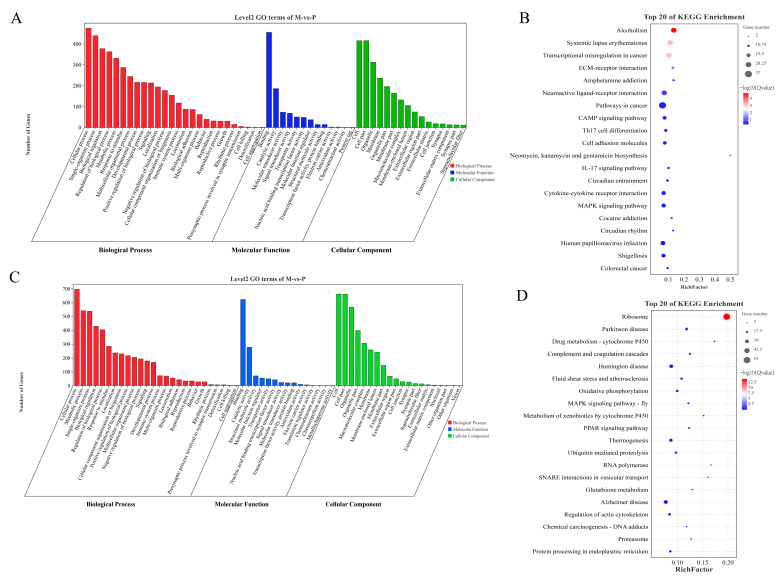
GO and KEGG analyses of DEMs. (**A**) GO terms significantly enriched by DEMs in Class-up. (**B**) Top 20 KEGG pathways significantly enriched by DEMs in Class-up. (**C**) GO terms significantly enriched by DEMs in Class-down. (**D**) Top 20 KEGG pathways significantly enriched by DEMs in Class-down.

The 783 up-regulated DEMs in Class-up were significantly enriched in 1480 GO entries, including 1264 GO-BP, 167 GO-MF, and 49 GO-CC (Table S3). In Figure 4A, the top 60 GO entries that were significantly enriched are depicted. The GO entry that was enriched to the greatest extent was cell adhesion (GO:0007155), and there were 17 other GO entries that pertained to the growth and development of muscles, including skeletal muscle cell differentiation (GO:0035914). There were 1081 down-regulated DEMs in Class-down that were significantly enriched in 470 GO entries (317 GO-BPs, 62 GO-MFs, and 91 GO-CCs) (Table S4). In Figure 4C, the top 60 GO entries that were significantly enriched are depicted. The intracellular ribonucleoprotein complex (GO:0030529) emerged as the most prominently enriched GO term, while muscle growth and development were represented by four GO entries, including muscle cell migration (GO:0014812). There were 36 KEGG pathways significantly enriched by the 738 up-regulated DEMs in Class-up, with the most significantly enriched pathway being alcoholism, followed by Systemic lupus erythematosus (Table S5). Furthermore, muscle growth and development were linked to five KEGG pathways, specifically ECM–receptor interaction, cAMP signaling pathway, MAPK signaling pathway, focal adhesion, and cell adhesion molecules. There were 25 KEGG pathways significantly enriched by the 1081 down-regulated DEMs in Class-down, with the ribosome pathway being the most significantly enriched (Table S6). Muscle growth and development were linked to two KEGG pathways, specifically the MAPK signaling pathway (fly) and the regulation of actin cytoskeleton. Figure 4B,D exhibit the notable enhancement in KEGG pathways in Class-up, along with the top 20 KEGG pathways that exhibited significant enrichment in Class-down.

### 2.5. Cis-/Trans-Regulation of the lncRNA Target Genes

**Figure 5:**
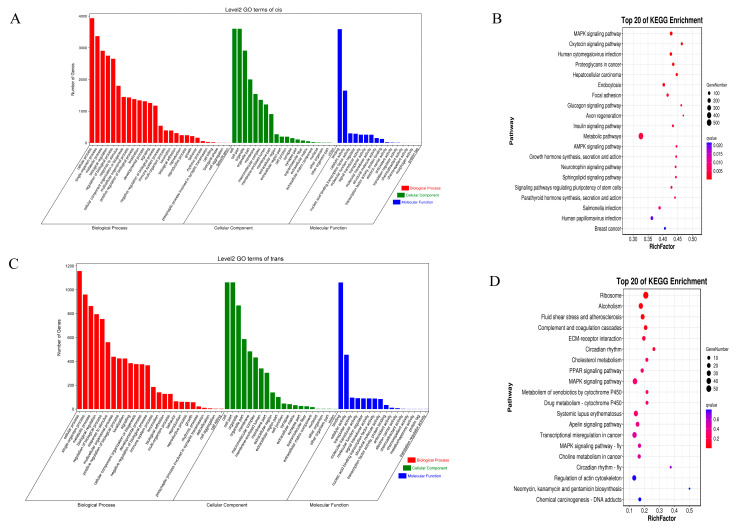
GO and KEGG analyses of cis- and trans-target genes of DELs. (**A**) The GO analysis of the top 60 cis-target genes with the corresponding categories. (**B**) KEGG pathways analysis of top 20 cis-target genes. (**C**) GO analysis of top 60 trans-target genes along with the categories of BP, CC, and MF. (**D**) The KEGG pathways of the top 20 trans-target genes.

Long non-coding RNA can control the expression of adjacent messenger RNA. By examining mRNA located within a 100 kb vicinity of the lncRNA and protein-coding genes, we successfully detected a total of 150 differentially expressed lncRNAs and 125 differentially expressed mRNAs (Table S7). Subsequently, the potential target genes of lncRNA were subjected to GO and KEGG analyses (Figure 5A,B). A total of 1224 GO entries (Table S8) were identified as significantly enriched (*p* < 0.05). Among them, the three most significantly enriched GO entries were cell (GO:0005623), cell part (GO:0044464), and intracellular (GO:0005622). Furthermore, we identified 90 KEGG pathways that exhibited significant enrichment (Table S9). Among these, the MAPK signaling pathway, the oxytocin signaling pathway, and Human cytomegalovirus infection emerged as the top three pathways with the highest level of enrichment.

Afterward, we detected 304 differentially expressed lncRNAs (DELs) and 1796 differentially expressed mRNAs (DEMs) by applying a threshold of r > ±0.95 to investigate the target genes of trans-lncRNA (Table S10). Figure 5C,D show the GO and KEGG results for DEL target genes. There were 1005 GO terms that showed significant enrichment (Table S11). Among these, ribosome (GO:0005840), the structural component of the ribosome (GO:0003735), and regulation of response to injury (GO:1903034) were the top three most significantly enriched GO entries. Out of 1005, 17 findings (*p* < 0.05) were associated with the enhancement and formation of muscles, including the migration of muscle cells. Additionally, we discovered 20 KEGG pathways that exhibited significant enrichment in KEGG (Table S12). Among these, the three most notably enriched pathways were ribosome, alcoholism, and fluid shear stress and atherosclerosis. Muscle growth and development were linked to four KEGG pathways, including the MAPK signaling pathway and ECM–receptor interaction.

### 2.6. Building a Network of Interactions between lncRNA and mRNA

**Figure 6:**
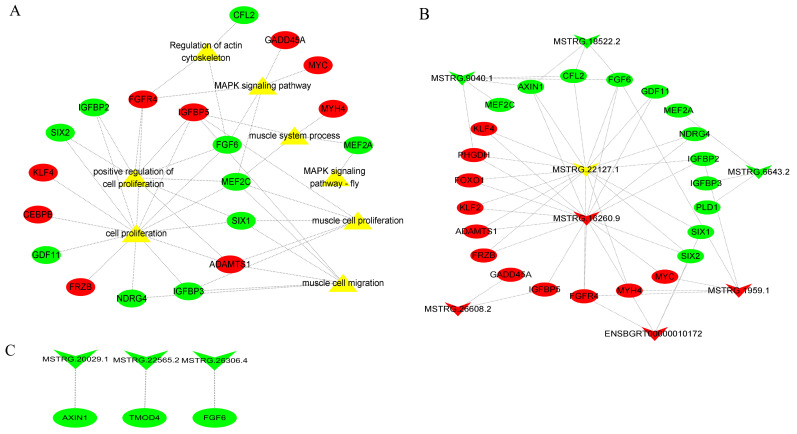
The DEL–DEM interaction network. (**A**) Network analysis of DE-lncRNA target genes and their enrichment pathways. (**B**) DEL and trans-target interaction network. The trans-target was also a DEM. (**C**) DEL and cis-target interaction network. The cis-target was also a DEM. The circle type and inverted-triangle type represent mRNA and lncRNA, respectively. Yellow represents the enriched signaling pathways; red represents up-regulation; and green represents down-regulation.

Here, we selected eight signaling pathways associated with muscle growth and development, among which the genes enriched in the MAPK signaling pathway were MEF2C, FGF6, GADD45A, FGFR4, and MYC. By conducting a literature search, we identified DEMs that impact muscle growth and development. We then obtained DELs and their corresponding target genes from cis and trans prediction results. Finally, we integrated these findings with DEMs from sequencing results to create a DEL–DEM interaction network. The network of DEL–DEM co-expression included 8 DELs and 55 trans-targets (Figure 6B), as well as 3 DELs and 3 cis-targets (Figure 6C). Several lncRNAs (MSTRG.16260.9, MSTRG.1959.1, ENSBGRT00000010172, and MSTRG.26608.2) were found to be up-regulated, while others (MSTRG.22127.1, MSTRG.9040.1, MSTRG.6643.2, and MSTRG.18522.2) were down-regulated, potentially linked to muscle growth and development. The results of the co-expression network indicated that certain DELs interacted with multiple DEMs. For instance, a specific DEL (MSTRG.16260.9 and MSTRG.22127.1) exhibited targeting associations with 16 genes associated with muscle growth and development. This suggests that lncRNA and mRNA are part of the essential non-coding/coding RNA that plays a crucial role in regulating the development of muscle growth.

### 2.7. Verification of Sequencing Results

**Figure 7:**
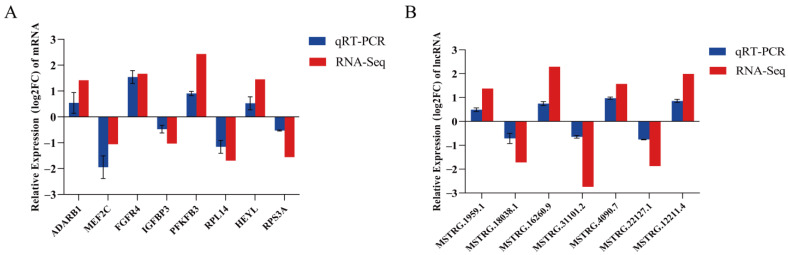
qRT-PCR results of DEMs and DELs. (**A**) Changes in mRNA expression in M and P treatment groups. (**B**) qRT-PCR validation of changes in lncRNA expression in M and P treatment groups. Three biological replicates were used for each group. The qRT-PCR data were determined with the 2^−ΔΔCt^ method using *GAPDH* as an internal reference. Validation data from mRNA and lncRNA sequencing results were further normalized to log_2_ (fold change). Data represent means ± standard errors.

We randomly chose seven lncRNAs and eight mRNAs from DEMs and DELs, respectively, and confirmed the precision of the sequencing findings with real-time quantitative PCR. The qPCR data in this study (Figure 7A,B) were found to be in agreement with the expression pattern observed in the sequencing data.

## 3. Discussion

The meat production and meat quality of Jeryak are superior to that of Gannan yak. With the aim of studying the mechanism of rapid growth in Jeryak, we explored the role of genes and lncRNA in the growth mechanism from the perspectives of myofiber characterization and transcriptome sequencing. Myofibers are the most basic units that make up a muscle, and their characteristics have a direct influence on the animal’s meat production performance and meat quality [8,9]. Meat tenderness was found to be greater when the muscle fibers had a smaller diameter and area [10]. In the present study, we found that the average muscle fiber area, average muscle fiber diameter, and average muscle fiber length of Jeryak were significantly smaller than those of Gannan yak. Moreover, the quality of muscle was found to be superior in cases where the proportion of slow-twitch muscle fibers exceeded that of fast-twitch muscle fibers [11,12]. We also observed that the area of fast muscle fiber was significantly increased in Gannan yaks compared with Jeryaks, while the area of slow muscle fibers exhibited a significant decrease relative to Jeryaks. These variations may serve as pivotal factors contributing to the disparities in post-partum meat production performance and meat quality between Gannan yaks and Jeryaks, thereby establishing the foundation for investigating their molecular regulatory mechanisms in this study.

In general, the regulation of muscle growth and development is orchestrated by core genes and signaling pathways. lncRNA, which is characterized by a length exceeding a certain value, has emerged as crucial a regulator of gene function and expression levels in this context [25,26,27,28]. According to recent research, lncRNA plays a crucial role in the regulation of skeletal muscle growth and development [29,30,31]. In the present study, a total of 304 DELs and 1819 DEMs were identified, among which 132 lncRNAs and 1081 mRNAs were found to be down-regulated, while 172 lncRNAs and 738 mRNAs were up-regulated. In particular, DEMs exhibited significant enrichment of GO terms associated with muscle growth and myoblast differentiation, including processes such as muscle cell migration. Based on the gene function analysis, we identified five up-regulated genes related to muscle development: *MYH4*, *FRZB*, *IGFBP5*, *MYH3*, and *FOXO1*. Additionally, three down-regulated genes associated with muscle development were also identified: *IGFBP3*, *SIXI*, and *MEF2C*.

*IGFBP3* and *IGFBP5* are part of the Insulin-like growth factor binding proteins (*IGFBPs*) family. During the process of myoblast differentiation, the inhibition of muscle cell differentiation occurs due to the presence of *IGFBPs*. These *IGFBPs* hinder the expression of *IGFs*, resulting in a decrease in protein synthesis. Moreover, the overexpression of *IGFBP5* can either enhance or hinder the *IGF*-mediated myoblast differentiation or survival by facilitating or impeding the action of *IGFs* [32,33]. Phosphorylation of the *FOXO* family occurs through the PI3K/AKT pathway in response to insulin, resulting in exclusion from the nucleus and subsequent inactivation [34]. Activation of *FOXO1* inhibits C2C12 differentiation and prevents AKT-induced myotube specialization [34]. Accili et al. similarly demonstrated that *FOXO1* exerts inhibitory effects on myofibroblast differentiation [35]. Furthermore, it has been demonstrated that *FOXO1* facilitates the degradation of muscle wasting [36]. Controlling the expression of genes related to the growth of skeletal muscles, such as *MYOG*, *MYHC*, *MYOD*, *IGF1R*, and *INSR*, is a vital function carried out by the Six1 gene [37,38]. *Six1* has a substantial impact on the growth and development of skeletal muscles from the embryonic stage to post-natal periods, as stated in this regulation [39,40]. Several studies carried out in zebrafish have demonstrated that the deletion of or reduction in *Six1* expression exerts a substantial impact on skeletal muscle development [41]. Additionally, it has been observed that knockdown of either *Six1a* or *Six1b* in zebrafish leads to a significant increase in apoptosis or cell death within embryonic myoblasts [39,40]. This highlights the critical role of *Six1* in promoting myoblast survival and normal function. *MEF2C* is commonly present in muscle tissues, particularly in skeletal and cardiac muscles [42]. *MEF2C* was the first DNA-binding factor identified to possess muscle characteristics and possesses the ability to regulate muscle growth by directly binding to promoters of muscle-specific genes such as *MCK* and *MYHC*. Additionally, *MEF2C* plays a crucial role in regulating skeletal myogenesis through direct interaction with *MYHC* [43].

The analysis results of the KEGG pathways revealed significant enrichment of DEMs in the Class-up category in 36 pathways. Among these pathways, five were found to be associated with growth and development, including ECM–receptor interaction, cAMP signaling pathway, and several others. In the Class-down category, DEMs were notably enhanced in 25 pathways related to muscle growth and development, such as the MAPK signaling pathway (fly) and regulation of actin cytoskeleton.

The metabolic adaptation of skeletal muscle to exercise is facilitated by the MAPK signaling pathway, which plays a significant role in this process. During exercise, the MAPK signaling pathway enhances insulin-dependent glucose uptake, increases oxidative metabolism, and promotes oxidative phosphorylation of mitochondria in muscle [44,45]. Moreover, this pathway is involved in the regulation of cell cycle arrest in mature myoblasts, a crucial step in initiating muscle differentiation [46]. In skeletal muscle cells, *MEF2C* activates the MAPK P38 pathway to induce differentiation of skeletal muscle [47]. In this study, we demonstrated that *MEF2C*, *GADD45A*, *FGF6*, *FGFR4*, and *MYC* may play crucial roles in regulating myogenesis in Gannan yak and Jeryak. These genes could be potential candidates for further enhancing the meat production performance of Gannan yak. The extracellular matrix (ECM), widely distributed in animal tissues, is a complex network synthesized and secreted by cells. It serves as both a physical scaffold for embedded cells and an essential support system for muscle differentiation [48,49]. Moreover, the ECM not only provides a structural framework for cell embedding but also regulates various cellular processes, such as growth, differentiation, migration, and survival. Additionally, it acts as a crucial reservoir of growth factors, facilitating intercellular signaling through attachment and release of numerous growth and signaling factors [50]. Focal adhesion plays a pivotal role in the regulation of skeletal muscle development, being intricately involved in several crucial signaling pathways, such as the WNT, MAPK, and PI3K-AKT intracellular pathways [51]. Moreover, it collaborates with the ECM to finely regulate intracellular pathways associated with cell movement, proliferation, and differentiation [52].

To better understand the potential functions of DELs, we performed GO and KEGG analyses on their cis-target and trans-target genes. In this study, we successfully identified 304 DELs and predicted their target genes, which were mainly enriched in GO entries related with muscle growth and development, particularly muscle structure development. These compelling findings strongly imply that DELs may exert a substantial influence on the intricate processes underlying muscle growth and development. Similarly, some lncRNAs have been recognized for their crucial involvement in the regulation of muscle growth and development. The KEGG analysis conducted on the target genes of DELs also revealed a significant association with key pathways, such as ECM–receptor interaction and the MAPK signaling pathway, which are known to play vital roles in muscle growth and development (*p* < 0.05). In addition, it has been observed that the Rap1 signaling pathway interacts with the β-adrenergic signaling pathway, thereby making a substantial contribution to skeletal muscle growth and development [53]. Kosuru et al. investigated the expression and localization of the Rap1 protein during different developmental stages of skeletal muscle and discovered that the Rap1 protein accumulates at the neuromuscular and tendon junctions in the early and late stages of myogenesis, respectively [54]. To screen lncRNAs and mRNAs that may be related to muscle growth and development more intuitively and deeply, we constructed a DEL–DEM co-expression network. Here, we selected eight signaling pathways associated with muscle growth and development, among which the genes enriched in the MAPK signaling pathway were *MEF2C*, *FGF6*, *GADD45A*, *FGFR4*, and *MYC*. The genes in these pathways that were present in the background were identified by intersecting them with the differentially expressed mRNAs that had been previously sequenced in the transcriptome [55]. These intersecting mRNAs and differential lncRNAs were used to construct an interaction network. In the interaction network, some DELs were found to interact with multiple DEMs. For example, two candidate DELs (MSTRG.16260.9 and MSTRG.22127.1) were found to have a targeting relationship with 16 genes related to muscle growth and development (*MYC*, *IGFBP5*, *IGFBP2*, *MYH4*, *FGF6, FGFR4*, etc.). We found that FGF (fibroblast growth factor) promotes proliferation and inhibits differentiation of skeletal muscle satellite cells [56,57]. *FGF6*, which is expressed at high levels, enhances the survival of C2C12 and primary myoblasts by activating Cyclin D1 through *FGFRI*. Down-regulation of *FGF6* leads to enhanced myoblast differentiation via *FGFR4*-dependent activation of ERK1/2, resulting in increased expression of *MYHC*, *MYOD*, and *MYOG* [58]. In our study, *FGF6* was found to be a down-regulated DEM that targets the up-regulated MSTRG.16260.9.

In addition, a large number of studies have found that lncRNA are important cis-acting regulatory elements that can regulate the expression of protein-coding genes (examples of such lncRNAs include Malat1 [59], Yam1 [60], and lncRNA-NEF [61]) through cis-acting. Cai et al. discovered a protein-coding gene (*Six1* gene) situated less than 100 kb away from lncRNA-*Six1* [62]. According to functional validation of lncRNA-Six1, it regulates the expression of the *Six1* gene and promotes cell proliferation and muscle growth [62]. A protein-coding gene (*FGF6*) was discovered to be situated less than 100 kb away from MSTRG.26306.4 and exhibited differential expression in Gannan yaks compared to Jeryaks. However, there are some limitations to our study. We focused on identifying differentially expressed genes in the constructed network but did not perform adequate functional analyses. Therefore, it is important to validate our findings with in vitro and in vivo functional studies. Further studies should include validation of the functions of MSTRG.16260.9 and MSTRG.22127.1 in trans-acting, as well as cis-acting MSTRG.26306.4 and the target gene *FGF6*, to explore their modes of action and specific roles in muscle growth and development.

## 4. Materials and Methods

### 4.1. Preparation of Animals and Tissues

The three Gannan yaks (Gannan male yak × Gannan female yak) and three Jeryaks (Jersey male cattle × Gannan female yaks) involved in this study were sourced from Hezuo City, Gannan Tibetan Autonomous Prefecture, Gansu Province. All animals were 4 years old and healthy. They were fed and watered ad libitum under identical conditions. The Experimental Animal Ethics Committee of Gansu Agricultural University approved the selection of adult bulls with comparable body weights, which were subsequently slaughtered in an abattoir. After slaughter, longest dorsal muscle samples were collected and stored in a refrigerator at −80 °C.

### 4.2. Staining of Muscle Tissue Using Hematoxylin and Eosin, as Well as Fluorescence Staining of Fast/Slow Muscle Fibers

To enhance the observation of the histological morphology of the muscle tissue, staining techniques, including HE staining and fluorescent staining for fast/slow muscle, were employed. The method by Zhang [63] was used to carry out HE staining. Initially, the muscle tissue was extracted from the fixative, followed by the preparation of paraffin sections. The only procedures conducted were embedding, sectioning, staining with hematoxylin and eosin, dehydration, mounting, and image acquisition. Next, the tissue sections were placed inside a container filled with EDTA antigen recovery buffer. Once cooled, the slides were immersed in PBS (pH 7.4) and cleansed by shaking them with a decolorizing shaker. Subsequently, a drop of blocking serum was introduced, followed by overnight application of a primary antibody and subsequently a secondary antibody. Subsequently, the nuclei were re-stained by adding DAPI staining solution drop by drop, followed by incubation of the cells at room temperature in the absence of light for a duration of 10 min. They were then cleaned using PBS. We added autofluorescence quencher, sealed the slides, observed the slices under a fluorescence microscope, and collected images. DAPI-stained cell nuclei appeared blue, while fast muscle fibers were indicated by the color red, and slow muscle fibers were indicated by the color green.

CaseViewer2.2 scanning browser software was utilized to select target regions for data analysis, with eight regions per slice. Afterward, the pictures were brought into image-pro Plus 6.0 software for image analysis to determine their diameter, area, and type. The statistical analyses were conducted utilizing SPSS 25.0 software.

### 4.3. Extraction of RNA, Construction of Library, and RNA Sequencing

Total RNA was extracted from muscle tissue using the Trizol reagent kit (Invitrogen, Carlsbad, CA, USA), and RNA integrity was precisely assessed using an Agilent 2100 Bioanalyzer (Agilent Technologies, Palo Alto, CA, USA). The analysis of RNA integrity and the detection of DNA contamination was conducted by employing electrophoresis on 1% agarose gel. Following the isolation of complete RNA, the ribosomal RNA was eliminated, and subsequently, the RNA was fragmented into smaller pieces. Random oligonucleotides were used as primers to synthesize the initial cDNA strand, while the second strand was synthesized using the DNA polymerase I system. Eventually, the process of PCR amplification was carried out, resulting in the acquisition of the library. PCR was used to amplify the libraries, which were then sequenced using Illumina NovaSeq 6000 by Gene Denovo Biotechnology (located in Guangzhou, China). The library-sequencing parameter was PE150 (paired-end 150 bp) (located in Guangzhou, China).

### 4.4. Data Quality Control and Reference Genome Alignment

For obtaining reads of excellent quality and free from impurities, it is recommended to utilize fastp (version 0.18.0) for filtering the reads. The settings are as follows: (1) eliminate reads that have adapters; (2) discard reads that have over 10% unidentified nucleotides (N); (3) eliminate low-quality reads that have more than 50% bases with low quality (Q value ≤ 20).

Using HiSAT2 software (version 2.1.0) [64], the high-quality clean reads of each sample were compared with the yak reference genome (LU_Bosgru_v3.0) to determine the remaining matches. Genome and gene alignment were used to evaluate all samples. The software Stringtie (version 1.3.4) [65] was utilized for transcript reconstruction.

### 4.5. lncRNA Identification and lncRNA Target Gene Prediction

Furthermore, for acquiring lncRNA data and conducting transcript screening, transcripts falling under categories “i”, “u”, “x”, and “o” were initially chosen. Subsequently, transcripts with lengths below 200 nt and exon counts less than 2 were eliminated. Following that, the software applications CPC (version 0.9-r2) [66], CNCI (version 2) [67], and Feelnc (version v0.2) [68] were utilized to predict the coding ability. Ultimately, the intersection of the outcomes from the three software applications was considered the identified lncRNA. There are two main approaches to predicting lncRNA targets: cis-acting and trans-acting [69]. To predict cis-acting effects, the co-location threshold was established as 100 kb in both the upstream and downstream regions of lncRNA in order to identify cis-target genes. Afterwards, the trans-target genes were filtered by examining the association between lncRNA and mRNA expression in the samples (Pearson correlation coefficient, r > 0.95).

### 4.6. Analysis of Genes with Differential Expression and Pathways

The Cuffdiff program [70] was utilized to conduct a comparative analysis of the 6 transcripts collectively, with FPKM values serving as indicators of gene or transcript expression levels. DESeq software (version 1.24.0) [71,72]. was utilized to normalize the counts of each sample. DEMs and DELs were identified by screening for mRNAs and lncRNAs with absolute log_2_FC values greater than 1 and FDR values less than 0.05.

To comprehend the role of lncRNA target genes, an analysis of GO (Gene Ontology) enrichment was conducted using the top GO. The hypergeometric distribution method was utilized to calculate significantly enriched GO entries, with a criterion of *p* < 0.05 for determining significant enrichment. Cluster profiler software (version 4.10.0) was used to conduct the pathway enrichment analysis of KEGG (Kyoto Encyclopedia of Genes and Genomes), with a significance criterion of *p* < 0.05.

### 4.7. DEL–DEM Co-Expression Network Construction

We anticipated lncRNA cis- and trans-target genes, intersected the forecast outcomes with mRNA sequencing outcomes, and subsequently examined the literature to filter the genes associated with muscle growth and development. Finally, we used Cytoscape (v3.6.0) software to map the target relationship between lncRNA and its target genes.

### 4.8. Verification of DEMs and DELs Using qRT-PCR

To verify the reliability of the sequencing results, 7 DELs and 8 DEMs were randomly selected for qRT-PCR. Primer 5.0 was utilized to create the primers, with *GAPDH* (glyceraldehyde-3-phosphate dehydrogenase) serving as the internal reference gene. The primer sequence details can be found in Table 3 and Table 4. First, total RNA was extracted from the samples using TRIzol reagent (Invitrogen, Carlsbad, CA, USA). Second, we used a reverse transcription kit (TransGen Biotech, Beijing, China) and a real-time fluorescence quantification kit (TransGen Biotech, Beijing, China) for cDNA synthesis and real-time fluorescence quantitative PCR. All qRT-PCR reactions were performed with an ABI Prism 7500 real-time PCR system (Thermo Fisher Scientific, Waltham, MA, USA). The total reaction system was 20 μL: 10 μL of 2× PerfectStart Green qPCR SuperMix, 0.4 μL of Passive Reference Dye (50×) (optional), 0.8 μL of primers, 2 μL of cDNA templates, and 6.8 μL of RNase-free water. The relative quantification of lncRNAs was determined utilizing the 2^−ΔΔCt^ approach and compared to the findings from transcriptome sequencing.

## 5. Conclusions

In the overview, we analyzed DELs and DEMs in the longest dorsal muscle tissues using Gannan yak as a control group. The identified DELs and DEMs were enriched in the pathways related to muscle growth and development. Based on these findings, we constructed a network of interactions between DELs and DEMs and discovered that MSTRG.16260.9 and MSTRG.22127.1 might play a role in regulating myogenesis in Gannan yak and Jeryak. These lncRNAs could potentially be utilized as candidates to further improve the production performance of yak.

## Figures and Tables

**Table 1:** Output statistics and quality assessment of cDNA library sequencing.

Sample	Raw Reads	Clean Reads	Low Quality (%)	Q20 (%)	Q30 (%)	GC Content (%)
M1	91,597,218	91,113,084	0.33	96.84	92.14	65.18
M2	90,970,934	90,561,790	0.27	96.93	92.40	65.54
M3	77,261,060	76,658,992	0.59	96.88	92.20	64.48
P1	91,772,588	91,351,006	0.25	96.95	92.24	72.24
P2	93,352,144	92,856,146	0.31	96.89	92.18	71.10
P3	90,267,324	89,794,208	0.30	96.68	91.77	72.18

**Table 2:** Clean reads and reference genome alignment results.

Sample	Total	Total Mapped (%)	Unique Mapped (%)	Multiple Mapped (%)
M1	68,296,270	55,971,679 (81.95%)	44,550,776 (65.23%)	11,420,903 (16.72%)
M2	68,471,616	55,259,551 (80.70%)	44,632,547 (65.18%)	10,627,004 (15.52%)
M3	59,586,390	49,279,578 (82.70%)	39,697,584 (66.62%)	9,581,994 (16.08%)
P1	50,960,524	36,586,723 (71.79%)	28,892,599 (56.70%)	7,694,124 (15.10%)
P2	56,700,506	41,707,068 (73.56%)	32,919,039 (58.06%)	8,788,029 (15.50%)
P3	51,396,228	36,426,543 (70.87%)	28,481,656 (55.42%)	7,944,887 (15.46%)

**Table 3:** lncRNA primers used in qRT-PCR.

lncRNA	Forward (5′→3′)	Reverse (5′→3′)
MSTRG.1959.1	GGAGGCTGAGACTGGAGGA	TGGGGTGTCGCTATGTTGC
MSTRG.16260.9	GTCCATCCATCCGCATCTC	CCTCCACTCTGACCATCCCT
MSTRG.4090.7	CTTGAAAAGTGGCTGTGGTT	ACGGATGGTGTCTGGAGGT
MSTRG.12211.4	CAGTAAAGTGTCTGCCTGTAATGC	TATCTCCCGAGTGGGTTGC
MSTRG.18038.1	ATTCGGGAAGGAGGTACAGG	TGGGGTTTACTCAAGGCACT
MSTRG.31101.2	GGCGGTCTACGGGTTATTC	CGCTTTCGTTCACAGGCTAA
MSTRG.22127.1	TGGCTGCTCTACTGTCCTCTG	CTGGTTGCCCCTGAATACG
GAPDH	AGTTCAACGGCACAGTCAAGG	ACCACATACTCAGCACCAGCA

**Table 4:** mRNA primers used in qRT-PCR.

mRNA	Forward (5′→3′)	Reverse (5′→3′)
ADARB1	AGCTGAACGAGATCAAGCCC	CTCGAACACCTGTCCGTTGA
FGFR4	CCTTGCTTCTGCACAACGTC	CCTGTCCATCCTTGAGCCAG
PFKFB3	AACCGTGTGCAGGATCACAT	GGTCCTTCAGGTTCTGCTCC
HEYL	ACTTCCGGAGCATCGGTTTT	TGCATAGCTGTTGAGGTGGG
MEF2C	CACTGGGAAACCCCAACCTT	AGCAGACCTGGTGAGTTTCG
IGFBP3	CGCTACAAGCGTTGTTGGAC	TGCTGTGGTCTTCTTCCGAC
RPL14	TTCAGGCGCTTCGTAGAGG	GCAGGTGCTTTCTGGGATG
RPS3A	GCGGTCGGCAAGAATAAG	TGGTGGCTCGTATCCATC
GAPDH	AGTTCAACGGCACAGTCAAGG	ACCACATACTCAGCACCAGCA

## Data Availability

The data presented in the study are deposited in the NCBI repository, https://www.ncbi.nlm.nih.gov/bioproject/PRJNA1023693, URL (accessed on 10 June 2023), accession number: PRJNA1023693.

## Supplementary Materials

The following supporting information can be downloaded at: https://www.mdpi.com/article/10.3390/ijms242316991/s1.
